# Patients developing inflammatory bowel disease have iron deficiency and lower plasma ferritin years before diagnosis: a nested case-control study

**DOI:** 10.1097/MEG.0000000000001816

**Published:** 2020-06-11

**Authors:** Lovisa Widbom, Kim Ekblom, Pontus Karling, Johan Hultdin

**Affiliations:** a Department of Medical Biosciences, Clinical Chemistry, Umeå University; b Department of Research and Development, Region Kronoberg, Växjö; c Department of Public Health and Clinical Medicine, Medicine, Umeå University, Umeå, Sweden

**Keywords:** Crohn’s disease, epidemiology, inflammatory bowel disease, iron deficiency, nutrition, ulcerative colitis

## Abstract

Iron deficiency is common among inflammatory bowel disease (IBD) patients, generally reported without comparisons with controls. The aim of this study was to analyse if iron deficiency was more common among those later developing IBD compared to matched controls in a prospective setting.

**Methods:**

We included 96 healthy subjects later developing IBD and 191 matched controls from the Northern Sweden Health and Disease Study. We analysed iron, ferritin, transferrin, and calculated transferrin saturation in plasma sampled at least 1 year prior to IBD diagnosis. Iron deficiency was defined as plasma ferritin <30 µg/L if C-reactive protein (CRP) was <3 mg/L. When CRP was >3 mg/L, iron deficiency could not be excluded if ferritin was <100 µg/L.

**Results:**

Iron deficiency could not be excluded among more male cases vs controls (25.0% vs 2.2%; *P* < 0.001), whereas with no differences for women (39.6% vs 35.3%; *P* = 0.538). Ferritin was lower among male IBD cases (*P* = 0.001) and for ulcerative colitis (*P* = 0.016 for males and 0.017 for females), but not for Crohn’s disease. Ferritin was associated with a lower risk for IBD and in the ulcerative colitis subgroup when using sex-based z-scores. Ferritin quartiles 2–4 had a 65% lower odds ratio for all IBD, ulcerative colitis, and Crohn’s disease in multivariable analysis.

**Conclusions:**

Lower ferritin was associated with higher risk for developing IBD in a prospective setting. Iron deficiency was more common among healthy males years later developing IBD compared to matched controls, but not among women.

## Abstract 2

Supplemental Digital Content is available in the text.

## Introduction

Inflammatory bowel diseases (IBDs) including ulcerative colitis and Crohn’s disease are chronic inflammatory disorders that involve an autoimmune inflammatory response directed against the gut mucosa by an unknown cause [1]. Anaemia in IBD is according to several previous studies commonly due to either iron deficiency anaemia (IDA), anaemia of chronic disease (ACD), or combined anaemia (IDA and ACD simultaneously) [2,3]. Iron deficiency can occur with or without anaemia. There are different definitions of iron deficiency [4,5]. In the European Crohn’s and Colitis Organisation (ECCO) guidelines from 2015 iron deficiency was defined as serum ferritin <30 µg/L in patients without signs of active disease, and in the case of inflammation, iron deficiency cannot be ruled out with ferritin <100 µg/L. IDA is defined as iron deficiency in combination with haemoglobin below 120 g/L for nonpregnant women and below 130 g/L for men [6].

Anaemia is common in active IBD, and the prevalence varies between 16 and 50% [7–10]. In a Scandinavian study, combined anaemia was most common, followed by ACD and IDA [3]. Several studies have reported the frequencies of iron deficiency without anaemia, both in active disease and at diagnosis, with a higher proportion among women.[11–13]

Most previous studies did not have any control group and thus were not able to compare their frequencies of iron deficiency or IDA with those of a healthy population. A study on 241 IBD patients in Switzerland reported a prevalence of uncategorized anaemia of 21.2% compared to 3.4% among 6074 first-time blood donors but did not report results for men and women separately [14]. In this study, iron deficiency was not assessed in all patients, as ferritin was analysed based on clinician decisions in cases with anaemia. We have not found any study comparing the prevalence of IDA in known IBD patients to matched controls. Only one study about iron deficiency has been conducted among nonsymptomatic persons [15]. In this study, 77 864 Korean men underwent screening with esophagogastroduodenoscopy and colonoscopy, and iron status was assessed in order to describe the correlation between Iron deficiency and different gastrointestinal disorders diagnosed. They showed that IBD was more common among asymptomatic people with IDA compared to people without IDA, though the number of IBD patients was very low (*n* = 5). To our knowledge, no prospective study has been performed to determine the occurrence of iron deficiency among healthy subjects later developing IBD.

Iron is involved in several complex biological processes, including, but not restricted to many enzymes [16], and the immune system,[17], which are not yet fully understood. The aim of this study was to test the hypothesis that iron status in plasma indicating low iron availability, Iron deficiency, or both are more common among those developing IBD in a prospective nested case-control study.

## Materials and methods

### Study population

The participants in this study were all part of the Northern Sweden Health and Disease Study (NSHDS). The NSHDS includes the Västerbotten intervention project (VIP) and the Mammography screening project (MA). VIP is an ongoing project collecting blood samples and survey data in conjunction with a health screening offered to all inhabitants in Västerbotten at 40, 50, and 60 years of age. During a visit to a primary healthcare centre, participants answer questions regarding lifestyle, including previous and active smoking, diet, body length, and weight, are measured and blood samples are collected for research. The project started in Norsjö, Sweden 1985, and since then, more than 150 000 questionnaires and blood samples have been collected. The participation rate has varied between 48 and67%.[18] The MA project collected blood samples and survey data simultaneously with mammography screening for women aged 18–82 years between the years 1995 and 2006 with about 54 000 blood samples obtained. All samples are stored at the Umeå University Medical Biobank.

Case identification was performed by searching for ICD-Codes (K50.1-9 or K51.1-9). An extensive review of medical records verified that the diagnoses were based on objective measures, including colonoscopy, computed tomography (CT), MRI, or biopsy. All cases were included in the NSHDS prior to diagnosis.

This nested case-control study initially included 121 patients and 239 controls. For each case, two randomly selected controls, matched by age, sex, time of blood sampling, and centre for blood sampling were selected. Cases diagnosed less than one year from data collection were excluded from the study. After exclusion, 96 patients with IBD (70 ulcerative colitis and 26 Crohn’s disease) and 191 matched controls were included in the study.

### Sample collection and handling

Blood samples were collected in Na-heparin tubes (10 mL), which were inverted 8–10 times directly after sampling. After 15 min at room temperature, the tubes were centrifuged at 1500G for 15 min. Plasma was frozen in aliquots within 1 h and stored at −80°C.

### Biochemical analyses

All plasma samples were analysed in batch in triplets with one case and matched controls in random order blinded to the technicians on a Cobas 8000 modular analyser (Roche Diagnostics GmbH, Mannheim, Germany). Reagents used were IRON Gen.2 (Cat No. 05169291 190), Ferritin (Cat No. 04491785 190), transferrin TRSF2 (Cat. No. 03015050 122), and CRPL3 (C No. 05172373190); all from Roche Diagnostics, Basel Switzerland. The lowest levels of detection were for iron 1 μmol/L, for ferritin 1 μg/L, for transferrin 0.20 g/L, and C-reactive protein (CRP) 3 mg/L. Iron was standardized to primary reference material SRM 937. Ferritin was calibrated to NIBSC Standard # 80/602. Transferrin was standardized to the Institute for Reference Materials and Measurements, BCR470/CRM470 (Reference Preparation for Proteins in Human Serum). CRP is traceable to CRM 470 (CRPL3 2011-01, V3). The total coefficients of variation were for iron 3% at levels 12 and 40 μmol/L, for ferritin 3.6% at levels 26.6 and 131.6 μg/L, for transferrin 4 and 6% at levels of 1.5 and 5 g/L, respectively, and for CRP 1.5 and 1.9% at levels of 8 and 47 mg/L, respectively.

Transferrin saturation (%) was calculated from iron and transferrin: P-Fe [µmol/L] × 100/(P-Transferrin [g/L] × 25.1).

### Statistical analyses

All statistical analyses were made using IBM SPSS statistical software package version 24.0 (IBM Corporation, New York, New York, USA).

Differences in fasting time were analysed using the Kruskal–Wallis test. Baseline characteristics were described as median and 25–75 percentiles for continuous variables and percent for proportions. Differences between cases and controls were analysed using the Mann–Whitney-U-test for continuous variables and the Chi-squared test for proportions.

Smoking was defined as current smoking, and former smokers were analysed as nonsmokers, all based on self-reported lifestyle survey data.

According to the ECCO guidelines from 2015, iron deficiency was defined as serum ferritin <30 µg/L, if CRP was <3 mg/L. When CRP was >3 mg/L, iron deficiency could not be excluded if ferritin was <100 µg/L [6].

For logistic regression, continuous iron parameters (iron, ferritin, transferrin, and transferrin saturation) were standardized as z-scores. z-scores were constructed, giving the mean the value zero, the value one is equal to one SD above mean, and minus one is equal to one SD below mean.

Quartiles were defined by dividing the controls into four equally sized groups separately for men and women, the quartile limits obtained were then used to divide all cases and controls into groups, with separate limits for men and women.

Conditional logistic regression was performed to assess the odds ratio (OR) of IBD for the different iron parameters. Adjustments were made for BMI smoking and CRP (as a marker for inflammation). The same models were used for both the z-scored continuous variables and for quartiles of the different iron parameters. A *P* value of less than 0.05 was regarded as a statistically significant.

A sensitivity analysis excluding subjects that used iron or multimineral supplementation during 14 days before sampling was performed, using the same logistic regression models as described above.

## Results

Ferritin was lower among cases who later developed IBD compared to controls (Table 1). When stratifying for sex, male cases had lower ferritin compared to controls, and no differences were seen for women. There were no differences for iron, transferrin, transferrin saturation, or BMI. Smoking was more common among the cases. Stratification for ulcerative colitis and Crohn’s disease showed no differences between cases and their matched controls in the whole group, although further stratification for sex showed that ferritin was lower among ulcerative colitis cases for both men and women. (Supplementary Table 1, Supplemental digital content 1, http://links.lww.com/EJGH/A556). There were no differences in fasting time between cases and controls, *P* = 0.902.

**Table 1.**
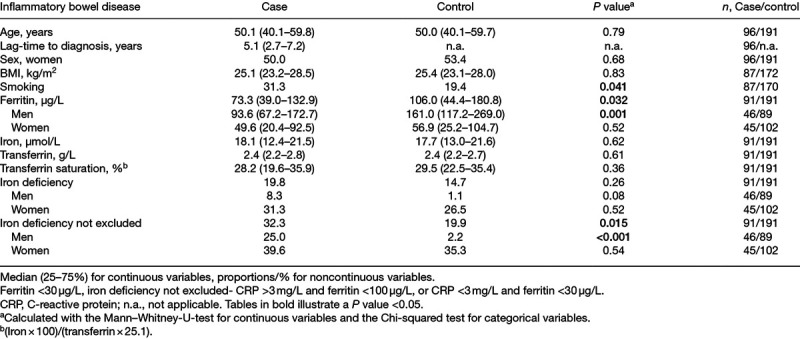
Baseline characteristics for inflammatory bowel disease cases and controls

For IBD, a ferritin-based model for iron deficiency showed no differences between cases and controls. When combining low ferritin with ferritin 30–100 µg/L among those with elevated CRP to a model, iron deficiency could not be excluded among a higher proportion of cases, compared to controls (Table 1). This was also evident when stratifying for the IBD subtype (Supplementary Table 1, Supplemental digital content 1, http://links.lww.com/EJGH/A556). This was seen for men only, no differences in iron deficiency frequencies were seen for women (Table 1 and Supplementary Table 1, Supplemental digital content 1, http://links.lww.com/EJGH/A556), and this was true even after stratifying the women into subgroups based on age over or under 52 years as a surrogate for menopausal status (data not shown).

### Univariable and multivariable analyses

Conditional logistic regression for sex-based z-scores for ferritin, iron, transferrin, and transferrin saturation as continuous variables is shown in Table 2. For IBD, an increase of 1 SD in ferritin was associated with a lower risk of developing IBD, in univariable analysis. When adjusting for sex, the ORs of z-scored variables were of the same magnitude and remained significant (data not shown). The lower risk was also seen after adjustments for CRP, BMI, and smoking. For ulcerative colitis, an increase in ferritin was associated with lower risk, even after adjustment for CRP. After further adjustments for BMI and smoking, the OR did not change but was no longer significant. No association was seen for the Crohn’s disease group. No associations were observed for iron, transferrin, or transferrin saturation in univariable analysis, and further adjustments showed no associations (data not shown).

**Table 2.**
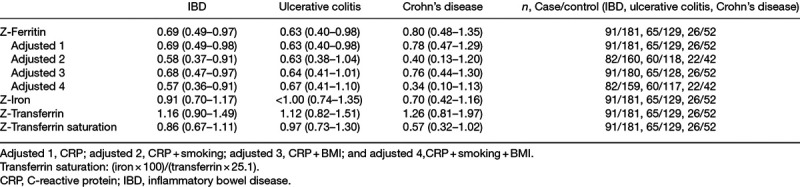
Conditional logistic regression, showing odds ratio and 95% confidence interval for sex-based Z-scores

There was a lower risk of developing IBD for ferritin quartiles 2–4 compared to controls, with a significant trend (Table 3), both in univariable analysis and after adjustments.

**Table 3.**
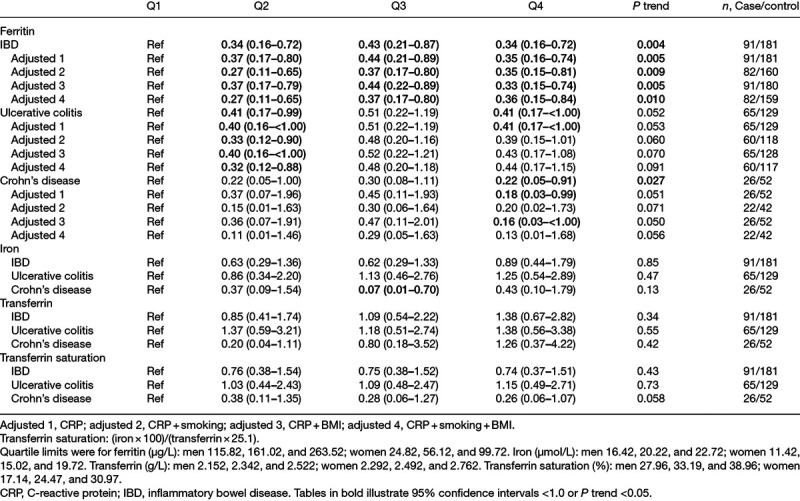
Conditional logistic regression, showing odds ratio and 95% confidence interval for quartiles of ferritin, iron, transferrin, and transferrin saturation

For ulcerative colitis, there was a lower risk seen in quartile 2, even after adjustments. A lower risk was also seen in quartile four in univariable analysis and after adjustment for CRP, although there was no significant trend. After further adjustments, the OR remained at the same magnitude but was no longer significant.

For Crohn’s disease, quartile four was associated with a lower risk of developing the disease in univariable analysis with a significant trend. This was also seen after adjustments for CRP and BMI, and after adjustment for smoking, this was no longer significant.

For iron quartiles, a lower risk was seen in quartile three for Crohn’s disease in univariable analysis only, and no other associations were observed. No associations were seen between quartiles of transferrin or transferrin saturation and risk for IBD, and further adjustments did not change the results (data not shown).

### Sensitivity analysis

Exclusion of subjects that used iron or multimineral supplementation during 14 days before sampling did not change baseline characteristics significantly (data not shown). For IBD and ulcerative colitis, the z-scored ORs for ferritin were significant in all models (Supplementary Table 2, Supplemental digital content 1, http://links.lww.com/EJGH/A556). There was also a significant risk reduction in the fourth ferritin quartile for all models, including a significant *P* for trend (Supplementary Table 3, Supplemental digital content 1, http://links.lww.com/EJGH/A556). The risk estimates did not change for other iron-related variables.

## Discussion

We show for the first time that iron deficiency is more common among healthy subjects who years later develop IBD compared to matched controls, especially among men.

People later developing IBD also have lower ferritin compared to controls. Adjusting for CRP, as a marker of inflammation, did not change the results. The associations were seen for both ulcerative colitis and Crohn’s disease when using ferritin quartiles. Continuous ferritin z-scores showed an association for IBD but only among ulcerative colitis.

A sensitivity analysis, excluding subjects that had used iron or multimineral supplementation 14 days before sampling, strengthened our findings: it revealed a significant OR for ulcerative colitis in the fourth ferritin quartile, and a significant trend in all models used. Also, for the z-scores, all models for ferritin in ulcerative colitis were significant. This study is the first to show that iron and multimineral supplementation may obscure risk associations for ferritin in ulcerative colitis, and thus, our findings emphasize the importance of adjusting for such supplementation.

As ferritin is a positive acute phase reactant, a possible early inflammation in cases later developing IBD would result in increased ferritin concentrations. Despite this, low ferritin was associated with increased risk for IBD. This may reflect lower iron availability early in the disease process, especially among men.

IBD has been associated with smoking status [19], and we have recently reported that never smokers have a lower risk for both ulcerative colitis and Crohn’s disease [20]. After adjusting for smoking status, the lower risk association seen for ferritin was still significant in all IBD cases vs controls. After stratification, the magnitude of the ORs for quartiles of ferritin did not change ulcerative colitis and Crohn’s disease subgroups, but they were no longer significant. This could be due to a lack of statistical power.

There are several possible causes of iron deficiency among IBD patients. It could be due to bleeding, exfoliation of cells, or both in patients with bowel mucosa damage. It could also be explained by decreased iron uptake from the upper small bowel (Crohn’s disease) and low dietary intake caused by the avoidance of food due to gastrointestinal symptoms.

The findings of a reduced risk through ferritin quartiles are similar to a previous prospective study on colorectal cancer [21], where higher quintiles of ferritin were associated with a decreased risk.

The risk associations were more pronounced in men. The variability in blood loss during menstruation and the subsequent changes in iron status may be a confounder, obscuring the possible risk associations with IBD in women.

Previous studies have reported the prevalence of iron deficiency in IBD, for example, one Canadian study on 280 IBD patients reported the prevalence of iron deficiency to 20% for Crohn’s disease and 27% for ulcerative colitis patients [22], compared to 20% iron deficiency in our material on IBD patients prior to diagnosis, 17% among future ulcerative colitis and 27% among future Crohn’s disease. This indicates that the changes seen in iron status among IBD patients are present years before diagnosis.

In women with IBD, previous studies have shown that iron deficiency is more prevalent compared to men. In a Spanish study on 104 patients, the prevalence of around 50% in women compared to 20% in men with IBD was seen [11]. A Swedish study of 373 patients with IBD reported a prevalence of iron deficiency and IDA of 6.8 and 6.2%, respectively, the total frequency of Iron deficiency with or without anaemia of 13% (8% in male and 16% in female IBD patients) [9]. This is consistent with our finding that iron deficiency is present in around 31.3% of women compared to approximately 8.3% of men prior to IBD diagnosis. Thus, iron deficiency is more prevalent in women compared to men. However, because our controls were matched, we can see that prior to diagnosis, women do not have a higher proportion of iron deficiency compared to controls, as is the case for men.

Ferritin levels <30 µg/L were reported in 24% of 150 IBD patients in a French study [23] and 25% in a Scandinavian study on 429 patients [24]. We found a slightly lower frequency of ferritin <30 µg/L, 19.8%, but our patients were healthy at inclusion. None of the studies mentioned above included any control subjects. In our study, iron deficiency was seen in 14.7% of controls. We also had a slightly higher median ferritin concentration, 73 µg/L, compared to 56 µg/L in Scandinavian patients with active disease [24]. Our study thus implicates that changes of ferritin and development of iron deficiency may occur already years ahead of diagnosis. There are a number of genome wide association studies reporting over 100 susceptible loci associated with IBD. No loci associated with iron metabolism have been implicated [25–29]. This might infer that changes in iron status in IBD are not due to genetic variations in iron metabolism, but somewhat attributable to gradual pathophysiological changes early in the disease process.

Iron deficiency and iron supplementation have been reported to alter the gut microbiota [30,31]. This, in combination with the known connection between the immune system reacting to the gut microbiota and development of IBD [32–34], may indicate that abnormalities in iron status also could contribute to the development of IBD. Interestingly, iron regulatory protein 2 and the HFE gene regulating iron uptake in the intestine have been associated with alterations of gut microbiota in mice [35]. As the latter gene is also an major histocompatibility complex class I molecule, there are possible interactions with the immune system [36]. Hepcidin, the major regulator of iron homeostasis and also influenced by HFE genotype, was early reported to have antimicrobial properties [37]. Further studies on the interactions of iron metabolism with gut microbiota and the immune system are needed.

Our findings emphasize the importance of knowing the aetiology of iron deficiency before starting treatment. Because iron deficiency is related to fatigue [38,39] and treatment with iron affects fatigue, without affecting the disease process in the intestine, this may result in a delayed diagnosis. It is also important to exclude causes of iron deficiency other than menstrual blood loss among women, not to postpone an IBD diagnosis.

There are limitations of our study; no haemoglobin was measured, and thus, anaemia could not be assessed. Because blood samples were collected in 40-, 50- and 60-year-old subjects, only late onset IBD could be studied. The results, therefore, are not generalizable to all patients with IBD; 22% of patients with Crohn’s disease and 28% of patients with ulcerative colitis get diagnosed after the age of 40 [40].

In addition, no data were collected at diagnosis; consequently, we could not estimate iron deficiency or markers of iron status at the time of diagnosis. Because the Crohn’s disease group is small, a connection between low ferritin or iron deficiency and developing Crohn’s disease cannot be ruled out.

Also, coeliac disease is a risk factor for iron deficiency and the prevalence of coeliac disease in the region of Västerbotten is approximately 0.5% [41]. None of the patients with IBD had comorbidity with coeliac disease; however, among the control subjects, this was not checked for.

The strengths of our study are that the samples were collected prospectively. We analyse data from subjects at least 1 year before diagnosis, thus reducing the effect of reverse causation. The cases are well characterized through extensive medical record reviews. Biobanking and preanalytical procedures were standardized, and cases were matched. All biochemical analyses were performed in batch. This study is also the first to our knowledge comparing both male and female IBD cases to matched controls. Another strength is that we also had data on CRP, as well as self-reported smoking, which made it possible to adjust for inflammation and smoking status. We also were able to account for the use of iron and multimineral supplementation during 14 days prior to sampling.

### Conclusion

We show for the first time in a prospective nested case-referent study that higher plasma ferritin in healthy subjects was associated with a lower risk for later developing IBD. Iron deficiency was more common among healthy male subjects who years later develop IBD compared to matched controls, but in women, iron deficiency was equally common in cases and controls. Iron deficiency could be an early marker for IBD, especially among men.

## Acknowledgements

We thank the participants of the Västerbotten intervention project and the Mammography screening project for their participation. We thank the Department of Biobank Research, at Umea University, the Västerbotten Intervention Program and Västerbotten County Council for providing data and samples and acknowledge the contribution from Biobank Sweden. We also thank the staff at Laboratory medicine, clinical chemistry Umeå University hospital for their skilful performance of the biochemical analyses.

This work was funded by the Västerbotten County Council, VLL-582981 and VLL-678111 and ‘Mag-Tarmfonden’. Biobank Sweden was supported by the Swedish Research Council (VR 2017-00650).

P.K. and J.H. conceived the study idea and designed the study; L.W. and P.K. collected the data; J.H. supervised biochemical analyses; all authors analysed the data, drafted the manuscript; and the final version of the manuscript was approved by all authors.

The study was approved by the regional ethical board, Umeå, Sweden (Dnr 06-024M, 2010-284-31M, and 2015-78-32M). Written informed consent was obtained from all participants before inclusion in the NSHDS.

## Conflicts of interest

There are no conflicts of interest.

## Supplementary Material


