# Supplementary material for: Patients developing inflammatory bowel disease have iron deficiency and lower plasma ferritin years before diagnosis: a nested case-control study
Source: Eur J Gastroenterol Hepatol. 2020 Jun 11;32(9):1147–53. doi: 10.1097/MEG.0000000000001816 (PMC7423531; doi:10.1097/MEG.0000000000001816)
Supplement: Supplementary file 1 [file ejgh-32-1147-s001.pdf]

Supplementary Table 1. Baseline characteristics for ulcerative colitis and Mb Crohn with matched controls. Median (25-75%) for continuous variables, proportions/% for non-continuous variables.

**Ulcerative colitis**	**Case**	**Control**	**p-value***	**n Case/Control**
Age, years	50.1 (40.0-59.8)	50.1 (40.1-59.7)	0.86	70/139
Lag-time to diagnosis, years	5.26 (2.66-7.23)	n.a.	n.a.	70/n.a.
Sex, women	61.4	54.7	0.77	70/139
BMI, kg/m^2	25.0 (23.2-27.5)	25.6 (23.1-27.8)	0.82	70/138
Smoking	30.0	20.1	0.16	65/128
Ferritin, µg/L	73.9 (40.6-141.3)	99.7 (44.8-180.2)	0.15	65/139
Men	101.8 (67.3-172.8)	161.0 (112.5-262.8)	**0.016**	32/63
Women	49.6 (22.4-92.5)	53.9 (24.9-99.0)	**0.017**	33/76
Iron, µmol/L	18.2 (14.7-22.0)	17.9 (12.5 -21.6)	0.88	65/139
Transferrin, g/L	2.41 (2.24-2.66)	2.43 (2.21-2.67)	0.92	65/139
Transferrin saturation, %**	28.8 (20.7-37.0)	29.1 (20.3-35.7)	0.93	65/139
ID	17.1	15.1	0.69	65/139
Men	8.8	0.0	0.06	32/63
Women	25.0	27.6	1.00	33/76
ID not excluded	28.6	20.1	0.14	65/139
Men	20.6	1.6	**0.003**	32/63
Women	36.1	35.5	0.87	33/76

**Mb Crohn**	**Case**	**Control**	**p-value***	**n Case/Control**
Age, years	50.2 (40.1-56.8)	50.0 (40.2-59.7)	0.86	26/52
Lag-time to diagnosis, years	4.76 (2.50-8.08)	n.a.	n.a.	26/n.a.
Sex, women	46.2	50.0	0.94	26/52
BMI, kg/m^2	26.1 (23.1-30.4)	25.3 (22.9-28.3)	0.43	26/52
Smoking	34.6	17.3	0.18	22/42
Ferritin, µg/L	68.7 (27.7-116.8)	116.6 (40.4-187.5)	0.082	26/52
Men	84.0 (64.1-162.5)	165.0 (123.0-276.3)	0.81	14/26
Women	40.5 (13.7-102.1)	64.3 (31.0-108.7)	0.39	12/26
Iron, µmol/L	17.4 (10.2-20.1)	17.6 (14.3-21.6)	0.22	26/52
Transferrin, g/L	2.47 (2.22-2.92)	2.36 (2.23-2.66)	0.37	26/52
Transferrin, saturation, %**	25.7 (16.7-31.1)	30.1 (23.5-35.0)	0.067	26/52
ID	26.9	13.5	0.25	26/52
Men	7.1	3.8	1.00	14/26
Women	50.0	23.1	0.20	12/26
ID not excluded	42.3	19.2	0.058	26/52
Men	35.7	3.8	**0.026**	14/26
Women	50.0	34.6	0.59	12/26

* Calculated with Mann-Whitney-U-test for continuous variables and Chi^2-test for categorical variables.

**(iron*100)/(transferrin*25.1).

n.a.: Not applicable. ID: iron deficiency, ferritin <30 µg/L, not excluded- CRP >3 mg/L and ferritin <100 µg/L, or CRP <3 mg/L and ferritin <30 µg/L.

Supplementary Table 2. Conditional logistic regression, showing odds ratio (OR) and 95% confidence interval (CI) for sex-based Z-scores. Supplement users excluded.

	IBD	UC	CD	n Case/Control (IBD, UC, CD)
**Z-Ferritin**	**0.64 (0.44-0.92)**	**0.54 (0.33-0.88)**	0.81 (0.49-1.34)	87/166, 62/118, 25/48
Adjusted 1	**0.63 (0.43-0.92)**	**0.54 (0.33-0.89)**	0.79 (0.48-1.30)	87/166, 62/118, 25/48
Adjusted 2	**0.50(0.30-0.82)**	**0.53 (0.30-0.93)**	0.40 (0.13-1.22)	78/145, 57/107, 21/38
Adjusted 3	**0.62 (0.42-0.91)**	**0.53 (0.31-0.90)**	0.77 (0.45-1.30)	87/165, 62/117, 25/48
Adjusted 4	**0.49 (0.29-0.82)**	**0.54 (0.30-0.98)**	0.35 (0.10-1.16)	78/144, 57/106, 21/38
**Z-Iron**	0.92 (0.70-1.20)	0.96 (0.70-1.31)	0.79 (0.46-1.35)	87/166, 62/118, 25/48
**Z-Transferrin**	1.23 (0.95-1.60)	1.16 (0.85-1.59)	1.38 (0.87-2.20)	87/166, 62/118, 25/48
**Z-Transferrin saturation**	0.84 (0.64-1.10)	0.91 (0.67-1.24)	0.62 (0.34-1.13)	87/166, 62/118, 25/48

Adjusted 1-CRP, Adjusted 2-CRP+smoking, Adjusted 3-CRP+BMI, Adjusted 4-CRP+smoking+BMI.

IBD- inflammatory bowel disease, UC-ulcerative colitis, CD-Crohn's disease, Transferrin saturation: (iron*100)/(transferrin*25.1).

Supplementary Table 3. Conditional logistic regression, showing odds ratio (OR) and 95% confidence interval (CI) for quartiles of ferritin, iron, transferrin, and transferrin saturation. Supplement users excluded.

	Q1	Q2	Q3	Q4	p-trend	n Case/Control
**Ferritin**						
IBD	Ref	**0.31 (0.14-0.69)**	**0.42 (0.20-0.88)**	**0.28 (0.13-0.61)**	**0.002**	87/166
Adjusted 1	Ref	**0.35 (0.16-0.77)**	**0.46 (0.22-0.98)**	**0.28 (0.12-0.64)**	**0.003**	87/166
Adjusted 2	Ref	**0.26 (0.10-0.64)**	**0.40 (0.18-0.89)**	**0.28 (0.11-0.69)**	**0.006**	78/145
Adjusted 3	Ref	**0.35 (0.16-0.77)**	**0.46 (0.22-0.97)**	**0.27 (0.11-0.64)**	**0.003**	87/165
Adjusted 4	Ref	**0.26 (0.10-0.64)**	**0.40 (0.18-0.89)**	**0.28 (0.11-0.72)**	**0.007**	78/144
UC	Ref	**0.36 (0.14-0.90)**	0.48 (0.20-1.16)	**0.31 (0.12-0.81)**	**0.019**	62/118
Adjusted 1	Ref	**0.37 (0.15-0.93)**	0.49 (0.20-1.19)	**0.32 (0.12-0.85)**	**0.027**	62/118
Adjusted 2	Ref	**0.31 (0.11-0.85)**	0.45 (0.18-1.15)	**0.30 (0.11-0.85)**	**0.031**	57/107
Adjusted 3	Ref	**0.37 (0.15-0.93)**	0.49 (0.20-1.19)	**0.32 (0.12-0.89)**	**0.032**	62/117
Adjusted 4	Ref	**0.31 (0.11-0.84)**	0.46 (0.18-1.17)	**0.33 (0.12-0.97)**	**0.047**	57/106
CD	Ref	0.23 (0.05-1.07)	0.33 (0.09-1.23)	**0.22 (0.05-0.90)**	**0.031**	25/48
Adjusted 1	Ref	0.35 (0.07-1.79)	0.45 (0.11-1.93)	**0.18 (0.03-0.99)**	0.051	25/48
Adjusted 2	Ref	0.14 (0.01-1.49)	0.32 (0.06-1.70)	0.17 (0.02-1.64)	0.072	21/38
Adjusted 3	Ref	0.33 (0.06-1.75)	0.47 (0.11-2.04)	**0.16 (0.03-0.99)**	**0.050***	25/48
Adjusted 4	Ref	0.10 (0.01-1.28)	0.32 (0.06-1.83)	0.11 (0.01-1.60)	0.059	21/38
**Iron**						
IBD	Ref	0.63 (0.28-1.420)	0.66 (0.30-1.43)	0.96 (0.47-1.97)	1.00	87/166
UC	Ref	0.81 (0.31-2.159)	1.12 (0.45-2.77)	1.24 (0.53-2.90)	0.50	62/118
CD	Ref	0.40 (0.09-1.814)	**0.09 (0.01-0.85)**	0.58 (0.13-2.55)	0.27	25/48
**Transferrin**						
IBD	Ref	0.93 (0.44-1.935)	1.26 (0.60-2.65)	1.66 (0.77-3.56)	0.17	87/166
UC	Ref	1.40 (0.59-3.340)	1.34 (0.55-3.24)	1.54 (0.59-3.98)	0.39	62/118
CD	Ref	0.26 (0.05-1.488)	0.91 (0.20-4.11)	1.68 (0.46-6.20)	0.24	25/48
**Transferrin saturation**						
IBD	Ref	0.81 (0.39-1.684)	0.79 (0.39-1.62)	0.76 (0.36-1.58)	0.46	87/166
UC	Ref	1.02 (0.43-2.455)	1.10 (0.48-2.53)	1.07 (0.44-2.59)	0.85	62/118

CD	Ref	0.46 (0.12-1.752)	0.32 (0.07-1.46)	0.33 (0.08-1.40)	0.11	25/48

Adjusted 1-CRP, Adjusted 2-CRP+smoking, Adjusted 3-CRP+BMI, Adjusted 4-CRP +smoking+BMI,
IBD- inflammatory bowel disease, UC-ulcerative colitis, CD-Crohn's disease, Transferrin saturation: (iron*100)/(transferrin*25.1).
Quartile limits were for Ferritin (µg/L): Men 115.82, 161.02 and 263.52, Women 24.82, 56.12 and 99.72. Iron (µmol/L): Men 16.42, 20.22 and 22.72, Women 11.42, 15.02 and 19.72. Transferrin (g/L): Men 2.152, 2.342 and 2.522, Women 2.292, 2.492 and 2.762.Transferrin saturation (%): Men 27.96, 33.19 and 38.96, Women 17.14, 24.47 and 30.97.
*0.49744
